# Home Environment, Bilingual Preschooler’s Receptive Mother Tongue Language Outcomes, and Social-Emotional and Behavioral Skills: One Stone for Two Birds?

**DOI:** 10.3389/fpsyg.2019.01640

**Published:** 2019-07-16

**Authors:** He Sun

**Affiliations:** National Institute of Education, Nanyang Technological University, Singapore, Singapore

**Keywords:** mother tongue language, home language environment, reading activities, social-emotional and behavioral skills, receptive Mandarin skills, harmonious bilingual development

## Abstract

The current study seeks to illustrate the relationships between child bilinguals’ mother tongue language (MTL) exposure and reading activities at home, children’s receptive MTL proficiency, and their socio-emotional and behavioral skills (SEBS). Data from 202 Singapore preschoolers (4–5 years old) who are learning English and Mandarin were analyzed. A parental questionnaire and standard Mandarin tests (i.e., receptive vocabulary, receptive grammar) were used to assess children’s Mandarin language-literacy environment at home, as well as their receptive language skills in Mandarin. Children’s SEBS were evaluated with the strengths and difficulties questionnaire (SDQ) (parental version). A series of variables which might influence SEBS and MTL proficiency (e.g., gender and SES) were controlled and SEMs were used to conduct data analysis. Results demonstrated that both Mandarin language and literacy environmental factors are related to children’s receptive language outcomes in Mandarin, while only literacy environmental factors associate with children’s difficulty level, and prosocial skills. This suggests that good parental support in bilingual children’s MTL literacy should be promoted not only for the sake of their early language development but also because of the potential benefits to their social emotional wellbeing.

## Introduction

In her pioneering papers, De Houwer (2009, 2015) put forward the concept of harmonious bilingual development and stressed its importance to children’s language and general well-being. As a feature of language contact at home, such harmony would bring children a positive experience with bilingualism and potential benefits in global judgments of life satisfaction. Parental input pattern, children’s language usage, and balanced language proficiency were highlighted as the key components of the concept. The author called for studies to examine the relations between children’s harmonious bilingual development and general wellbeing with large group studies, and eventually “serve the people we study more than we have been doing so far” (2015, p. 179). In many bilingual societies, the key to achieving such harmonious and balanced bilingual development probably lies in the environment of children’s mother tongue language (MTL) learning, as children are usually exposed to the input-rich societal language environment while their MTL learning setting is much less favorable (i.e., Sun et al., 2018b). A child’s MTL can refer to either a home or a heritage language (Cummins, 2001; Montrul, 2010). In the former case, the child uses the MTL at home, and in the latter case, the child may simply have a “heritage connection to the language” but not use it actively (Cummins, 2005, 586). MTL is known to be difficult to maintain in the young generation, as utilitarian parents might perceive the social functions of MTL (e.g., cultural and value preservation) as much less important than the communicative functions of the societal dominant language (e.g., English in the United States) in business, government, school, and media. Singapore is an example of such a society.

Renowned as a “melting pot” of diverse cultures, the Singapore population comprises 74.3% Chinese, 13.4% Malays, 9.0% Indians, and 3.2% other ethnic groups (Singapore Department of Statistics, 2017). Since 1965, English has been promoted in Singapore as the societal language with the goal of facilitating inter-ethnic communication and business with the world at large. Such a language policy has resulted in language shift, as “English is increasingly becoming the mother tongue for more and more Singaporeans, and their ethnic languages are technically more like second languages” (Cavallaro and Ng, 2014, 36). There has been found to be a substantial difference in language use and proficiency in English and the official MTLs (i.e., Mandarin, Malay, and Tamil) in preschoolers (Sun et al., 2018b). To mitigate the issue of declining MTL usage, the Singapore government initiated education programs to encourage MTL usage at home and at school, in an attempt to anchor children to their ethnic, and cultural traditions (Gopinathan, 2004). Despite such an endeavor, the trend of shifting home language to English is difficult to combat, due to parents’ utilitarian focus on English (Li and Tan, 2016). This trend has been demonstrated by reports of the Ministry of Education, where 49% children from the primary cohort were from English dominant families, and the figure increased to 59% in 2009 (Oon and Kor, 2009) and to 61% in 2011 (Goh and Ng, 2015).

To fundamentally change parents’ attitude, they should be explicitly informed about the advantage of children’s MTL acquisition. MTL maintenance has been well-documented to promote the acquisition of bilingual children’s other languages and to support their academic achievement (Oller and Jarmulowicz, 2007). Besides linguistic and academic outcomes, MTL is also important for sustaining cultural identity (Gudykunst and Ting-Toomey, 1990; Sun et al., 2018b), transmitting family values, fostering intimacy, and promoting attachment (Yu, 2013), which is pertinent to a child’s social-emotional wellbeing. Children’s social-emotional and behavioral skills are critical as they facilitate children’s adaptation to new environments (e.g., school transition) and help them to deal with everyday challenges. As its name indicates, social-emotional, and behavioral skills is a multifaceted concept, referring to a person’s general social competence, such as peer relationships, emotional recognition and regulation, and the ability to adjust to various contexts (Vahedi et al., 2012). Better social-emotional and behavioral skills have been found to relate to better academic performance and healthier wellbeing, while the lack of social competence has been associated with personal, social, and academic difficulties (Eisenberg, 2006). Our study aims to examine the association of specific home and literacy practice with both Mandarin acquisition and social-emotional and behavioral skills in English-Chinese preschoolers. The findings of the study might be not only useful to raise parents’ awareness of the benefits of MTL acquisition, but also to inform them of how to exert their influence on their child’s MTL learning via language contact, and literacy activities. The following section provides a literature review of the specific home language and literacy factors.

### Language and Literacy Exposure at Home

Different terminologies have been used to categorize child bilinguals and their learning settings (e.g., simultaneous and sequential bilinguals; Baker and Wright, 2017). Due to the heterogeneous nature of the MTL learners in terms of their bilingual input structure at home and the onset ages of their two languages, our participants could be generally called “dual language learners” (Hammer et al., 2014), which refers to children who are exposed to two languages in their early childhood. Both children who have input from two languages since birth and who are exposed to their second language at preschool are considered bilinguals in the current study. Research on factors that influence child bilinguals’ MTL acquisition *per se* or their social-emotional wellbeing is limited (but see Han, 2010; Sun et al., 2018c). Therefore, in the following literature review, we also include findings on child bilinguals’ second language learning. Both language exposure and literacy activities at home have been found to intimately and positively shape child bilinguals’ MTL abilities (Hoff et al., 2012) and social-emotional wellbeing (Sun et al., 2018c).

### Home Language Exposure

Language input at home (i.e., quantity and quality) has been demonstrated to be fundamental in modulating children’s MTL acquisition (Pearson, 2007; Li and Tan, 2016; Sun et al., 2018b), influencing children’s vocabulary (Scheele et al., 2010; Dixon et al., 2012; Sun et al., 2018a), and grammar (Paradis et al., 2011). To take vocabulary learning as an example, the amount of exposure to Spanish experienced by toddlers and preschoolers at home has been found to be positively related to English-Spanish bilingual children’s Spanish vocabulary in the United States (Hammer et al., 2009; Hoff et al., 2012). Input quantity could be estimated by the frequency that families and friends speak to the child in that language (i.e., current input pattern), and the length of the exposure since the child’s birth (i.e., cumulative input). Input quality could be operationalized in terms of the richness of the materials and the intensity (Jia and Aaronson, 2003). Higher maternal language proficiency (Chondrogianni and Marinis, 2011) and more social time with native speakers (Jia and Aaronson, 2003) are found to facilitate acquisition rates and general outcomes of vocabulary and grammar in bilingual children’s second language.

Besides hearing the MTL, children also need to use it to improve their proficiency in the language (Hammer et al., 2012; Lewis et al., 2016). This can be explained by Swain’s (1993) output hypothesis, which posits that the act of producing the language allows the person to be consciously aware of their language abilities, therefore facilitating reflection of self, and other language factors. This has been supported by a Singaporean study (Sun et al., 2018b), where bilingual’s language output in the three ethnic groups significantly predicted both the vocabulary sizes of the societal dominant language and the MTL. The relationship regarding these home language factors can be visualized as the input-proficiency-use cycle by Pearson (2007), where greater language input leads to greater proficiency, which will then increase child’s self-efficacy in using the language. The cycle will be self-sustaining once the language output from the child can elicit greater input from the environment.

Mother tongue language exposure may also influence their social-emotional skills (Han, 2010; Sun et al., 2018c). For instance, Han (2010) targeted Spanish-English bilinguals’ family language use and children’s English proficiency through teacher report and school entry tests to categorize these children’s language dominance. Balanced and sufficient language use was found to contribute to the development of children’s social-emotional and behavioral skills. Sun et al. (2018b) specified the contribution of input and output factors in their study on 805 Singapore bilingual pre-schoolers, and found that after controlling for gender, non-verbal intelligence, SES, and emotional recognition, those children who had larger bilingual receptive vocabularies and had frequently spoken both English and MTL for a longer time had better social-emotional and behavioral skills. Larger vocabulary size enables children to comprehend messages better and allows them to respond to others in an appropriate manner (Raver and Knitzer, 2002; Collins et al., 2011), while longer bilingual language practice may familiarize children with using language as a cultural artifact to regulate their behavior and establish better relationships at school and at home (Lantolf and Thorne, 2007). Through collaborative conversations, children are able to form inquiries and raise questions to bridge their knowledge gap. They also need to respond to questions and coordinate with other interlocutors to find the optimal answers.

### Home Literacy Activities

As input quantity, quality, and language output at home provide an overall indicator of child bilingual’s MTL language contact at home, literacy activities and literacy material availability provide information about the specific language and literacy characteristics in the home setting (Lewis et al., 2016). Home literacy exposure has generally been defined as the totality of socially constructed practices that implement skills dealing with print and non-print based texts and in critical thinking (Kahn and Kellner, 2005), comprising a plethora of attitudes, resources, and practices in the home that can influence children’s literacy practices and development (Burgess et al., 2002). Among the specific factors, reading activities and available materials have been repeatedly found to facilitate child bilingual’s MTL vocabulary, and oral abilities (Farver et al., 2013; Sun et al., 2018b).

Book reading and library visits can evoke children’s interest in reading and facilitate their development of emergent language and literacy skills (Lewis et al., 2016). During book reading, children may get access to a variety of words and challenging questions, which provide them with chances to acquire novel words, sentence structures, and concepts (Hoff, 2006). A series of studies have shown a link between the frequency of home literacy activities and child bilinguals’ L2 learning. For instance, Paradis (2011) investigated 69 immigrant children’s L2 learning in Canada and found that their home literacy activities significantly predicted verb morphology and vocabulary scores in their L2. This finding is in line with Scheele et al. (2010) findings, which revealed significant associations between home literacy activities like reading, storytelling in L2, and receptive and oral vocabulary outcomes in Moroccan-Dutch and Turkish-Dutch learning children. Not only the frequency of literacy activities, but also the availability of literacy materials at home matters. Usually indicated by the number of books at home, the availability of literacy materials at home implies the number of possible opportunities for children to participate in literacy and language-related activities (Hess and Holloway, 1984). Such availability may engage children in language-enriched activities that promote their receptive and productive language abilities (Burgess, 2005). Researchers have found a consistent relation between the availability of literacy materials (i.e., the number of children’s books), and monolingual children’s vocabulary size (Johnson et al., 2008). Findings from child bilingual’s MTL learning are mixed. Some have found the same positive relations (Lewis et al., 2016) as shown in the monolingual population, while others have observed no such relations (Farver et al., 2013). This is probably due to the fact that many of the studies have not specified whether the reading activities and materials are devoted to the children’s societal language or MTL (e.g., Lewis et al., 2016). The current study would like to examine this association by directly linking the child bilingual’s MTL reading activities and materials with children’s MTL outcomes.

Similar to home language exposure, home literacy activities have also been related to socio-emotional and behavioral skills (SEBS) in children (Farver et al., 2006; Aram and Aviram, 2009; Aram et al., 2017). For instance, Farver et al. (2006) studied the relationship between literacy activities like library visits and joint book readings with children, and bilingual children’s vocabulary and social functioning scores in low SES families at the Latino Head Start preschool program. Upon controlling for children’s age, parents’ education, family size, and parents’ literacy habits, results demonstrate that parental involvement in joint literacy activities is strongly correlated with both the vocabulary and social functioning scores of their children, and that this relationship is mediated by children’s interest in literacy. During literacy activities such as shared book reading, child bilinguals can develop basic social skills, such as turn-taking, self-expression, and self-regulation in addition to improving their oral vocabulary (Farver et al., 2006). When using books with social-emotional content, adults could initiate conversations on developing an understanding of certain prosocial skills, such as cooperation and sharing (Ziv et al., 2013). Such content could present models for children to solve problems and to interact with others, and potentially connect children emotionally with the characters in the story.

### Other Influential Factors on MTL Acquisition and Social-Emotional Skills

Despite their importance, home language and literacy factors should not be isolated from other influential factors to be examined. Child individual differences, such as gender and social economic status (SES) could possibly influence linguistic and social-emotional outcomes and should be considered simultaneously.

Research on the effect of gender on child bilinguals’ MTL acquisition remains inconclusive. While certain studies demonstrate that girls outperform boys in early MTL vocabulary performance (Eriksson et al., 2012) probably because they stay at home longer (Portes and Rumbaut, 2001), others could not confirm this conclusion (Dixon et al., 2012). The current study will examine the significance of this factor with our sample bearing the societal and cultural context in mind. On the other hand, gender seems to have a clearer link to social-emotional outcomes. It has been shown to predict level of externalizing or internalizing behaviors using varied socio-emotional measures, where boys were observed to exhibit higher levels of externalizing behaviors than girls (Ebert et al., 2013; Ren et al., 2016). Specifically, boys were reported to score higher in the Difficulties subscale of the strengths and difficulties questionnaire (SDQ) as opposed to girls, while girls scored higher in the Prosocial subscale in the Danish population (Niclasen et al., 2012), as well as in the Singapore context (Bull et al., 2016; Sun et al., 2018c).

Another factor that has been frequently studied is SES, predominantly operationalized by maternal education in the current research literature. SES has been long established to predict early language, academic and social development (McLoyd, 1998). This can be explained by the investment model, which states that the amount of time, effort, and money parents invest in their children can correspond to the number of potential avenues for improving linguistic, cognitive and emergent literacy skills (Dickinson and Tabors, 2001; Hartas, 2011). Moreover, socio-economic disadvantages can have adverse implications for parent-child relationships through higher family stress and emotional risks, therefore impacting the social and emotional competencies of the child (Winer and Thompson, 2013).

Besides gender and SES, cognitive factors like phonological short-term memory and non-verbal intelligence were also found to affect child bilinguals’ MTL development. Phonological short-term memory is essential for children to store and manipulate novel linguistic input to facilitate processing and organization of linguistic input in semantic memory (Paradis, 2011), while non-verbal memory allows children to reorganize, and analyze novel linguistic patterns to aid language acquisition (Daller and Ongun, 2017).

### The Current Study

To sum up, previous studies reveal that both children’s home language and literacy exposure could affect child bilinguals’ MTL learning and social-emotional and behavioral skills. Nevertheless, many of them focus either on language exposure or literacy activities to examine children’s MTL or social competence. Therefore, it remains unknown which of the specific environmental factors relate to children’s learning in both aspects. As home language and literacy factors are usually weakly or moderately correlated, it would be necessary to include both to pinpoint the specific practice we would encourage parents to conduct at home. Such findings are particularly crucial to countries like Singapore, where explicit knowledge about the reason and the approach to creating a harmonious MTL environment at home may greatly promote parents’ awareness and pedagogical practice. Two research questions are addressed.

(1) Which Mandarin home language and literacy factors can predict children’s receptive language skills in Mandarin? Children’s receptive language skills have been operationalized as Mandarin receptive vocabulary and Mandarin receptive grammar. We focused on children’s receptive language skills in the current study because previous studies indicated that their productive language skills would probably be limited during the first two years of the language instruction (Aarts and Ronde, 2006; Unsworth et al., 2015).

(2) Which Mandarin home language and literacy factors can predict children’s social-emotional and behavioral skills? Children’s SEWB has been operationalized as prosocial skills and difficulty level.

## Materials and Methods

### Participants

Two hundred and two young English-Chinese bilingual preschoolers in Kindergarten 1 (4–5 years old; 89 boys and 112 girls) were recruited from 21 preschools in Singapore to participate in this study. The majority of the participants were from PAP Community Foundation, which is the largest operator of kindergarten and childcare centers in Singapore. We recruited participants based on information provided by teachers and parents and there were two criteria for participant selection: firstly, children should be Mandarin-English bilingual language learners. Those who were exposed to more than two languages at home or recently emigrated from China were excluded. Secondly, participants should have no history of developmental delays or impairment. Socio-economic status varied among children, but most of them were from middle-class families, whose family income was well above the relative poverty line of the country (>S$2500, Donaldson et al., 2013). The average household income per month was between S$7500 to S$7999. There were 20 income options in the parental questionnaire, ranging from “Below 1,000” to “10,000 and over,” with S$500 increment for each higher level (*M* = 15.01, *SD* = 5.07, range = 0–19). On average, parents possessed a polytechnic or bachelor’s degree as the highest degree (e.g., mother’s education; *M* = 5.34, *SD* = 1.28, range = 2–8, ranging from “No qualification” to “Doctorate degree”).

### Data Collection and Measures

The author of this paper obtained ethics approval from the University’s institutional review board. Parents gave their informed consent through consent forms disseminated from preschools. Prior to the language assessments, children also gave their assent to complete the tasks through forms. Children’s Mandarin and English competencies (i.e., vocabulary and grammar) and cognitive capacities were assessed with standardized measures, while information relating to their bilingual environment at home were collected through parental questionnaire. The following section provides details of the measures and the questionnaire.

#### Mandarin and English Receptive Vocabulary

To measure children’s Mandarin and English receptive vocabulary, we used the bilingual language assessment battery (BLAB) (Rickard-Liow et al., unpublished), a standardized test similar to the peabody picture vocabulary test II (Dunn and Dunn, 2007). The auditory-picture matching task was developed in Singapore and has been reported to have good reliability in the context of Singapore within the original bilingual norming sample (alphas of 0.75 – 0.77) (Rickard-Liow et al., unpublished). Children were presented with four pictures while listening to recorded words in the program. They were then asked to select one picture out of four that best conveyed the understanding of the word mentioned to them. There were 3 practice trials and 80 test trials in total.

#### Mandarin and English Receptive Grammar

The Mandarin Grammar Receptive Test (MGRT; Bak, 2012, unpublished manuscript), and the English Test for Reception of Grammar Version 2 (TROG; Bishop, 2003) were used to measure children’s receptive grammar knowledge. Similar to procedures in the BLAB, children saw four images and heard a spoken sentence at the same time. They had to select one image out of the four pictures based on their understanding of the sentence heard. There were 3 practice trials and 60 test trials in total. Both MRGT and TROG have demonstrated good external validity and internal reliability (e.g., MRGT: Cronbach’s α = 0.75).

#### Social-Emotional Skills

Parents were asked to rate their children’s social-emotional and behavioral skills on the strengths and difficulties questionnaire (SDQ; Goodman, 1997). The SDQ is a screening questionnaire measuring children’s social-emotional wellbeing via children’s behavioral functioning. The SDQ has been validated and used across many different linguistic and cultural contexts (Croft et al., 2015), including Singapore (Bull et al., 2016). The subscales of this assessment tool have significant correlations with clinical diagnoses of related behavioral issues (Downs et al., 2012), and are useful in research contexts as well. SDQ is considered the most appropriate tool for assessing children’s social and emotional wellbeing by some researchers (Australian Institute of Health and Welfare, 2012). The SDQ has 25 items to characterize the socio-emotional skills of children ages two to sixteen. There are five subscales with five items each, namely (1) emotional symptoms, (2) conduct problems, (3) hyperactivity or inattention, (4) peer-relationship problems, and (5) prosocial behavior. Each item was rated on a 0 (not true) to 2 (certainly true) scale, and the sum score of each subscale ranges from 0 to 10, with higher scores indicating increased social-emotional and behavioral difficulties (scales 1–4) or better prosocial skills (scale 5). A Total Difficulties score (“Difficulty”) was generated by summing the first four subscales and a prosocial skills score (“Prosocial”) was derived based on the last subscale. The validity and reliability of parent SDQ have been reported as satisfactory in some review studies, with good pooled internal consistency for the total difficulties score (parent: α = 0.80; *n* = 53,691) (Stone et al., 2010).

#### Non-verbal Intelligence

The ravens colored progressive matrices (CPM) test (Raven and Rust, 2004) was used as a non-verbal measure of children’s general cognitive ability, and consists of three sections (A, AB, and B) of twelve items each. Children were provided an incomplete puzzle and asked to choose one out of six pieces to complete the puzzle. The items are arranged to allow for the assessment of consistency in the children’s reasoning using analogy and inference skills. The Ravens CPM test has been extensively used across a variety of settings worldwide as a culture-fair instrument of non-verbal intelligence.

#### Phonological Working Memory

To measure phonological short-term memory, the digit span and non-word repetition sub-tests of the comprehensive test of phonological processing (CTOPP; Wagner et al., 1999) were administered. The two tests comprise a list of digits or non-words in English, and participants were asked to repeat and pronounce what they heard on the computer. There were 21 and 18 trials respectively, for each subtest, and each subsequent trial increased in difficulty as the length of the digits and non-words increased in length. The tests were terminated when the child gave five consecutive incorrect responses.

#### Parental Questionnaire

A parental questionnaire was administered that included items adapted from previous related studies to explore children’s home language exposure (De Houwer, 2007; Sun et al., 2016) and literacy environment (Sun et al., 2018b). Compared with the prior questionnaires, the current version focused on children’s Mandarin, and English learning environment at home in particular. The first section of the questionnaire focused mainly on home language factors (input quantity, output), and the second section on home reading activities (e.g., reading frequency). Current language input and output were measured by the amount of time family members and friends speak in Mandarin to children and vice versa, whereas cumulative output was measured by the age at which the child starts to use Mandarin. For home reading measures, parents were asked about the number of Mandarin books at home using a scale ranging from 0 to 6 (0 = None, 1 = 1–10, 2 = 10–30, 3 = 30–60, 4 = 60–90, 5 = 90–120, and 6 = More). They were also asked to report the occurrence of joint literacy activities like library visits (Never, Once every 2 weeks, Once a week, Twice a week, 4 times each week, more than 4 times each week), and joint reading days at home per week.

### Data Analysis

Structural equation modeling (SEM; IBM SPSS AMOS 25) was used to assess the postulated relationships. SEM is a multivariate latent trait technique that has been widely used in the social sciences. It permits evaluation of multiple causal and correlational assumptions. The minimum sample size for SEM analysis should be 200 (Kline, 2011; Wolf et al., 2013). Four measures of fit for our models were selected based on the literature (Klem, 2000). Besides Chi-square, we also report Tucker and Lewis’s fit index (TLI), comparative fit index (CFI), and the root mean square error of approximation (RMSEA). A non-significant Chi-square implies good model fit as it indicates that the theoretical model does not differ significantly from the data-driven model. However, this is difficult to achieve, as Chi-square is sensitive to sample size. TLI and CFI values would not be affected by sample size, and higher values of these two indicators (>=0.9) suggest good model fit (Aryadoust and Liu, 2015). In contrast, smaller RMSEA values (<=0.06) indicate good model fit (Kenny and McCoach, 2003).

## Results

### Descriptive Statistics and Bivariate Correlations

The descriptive statistics of predictors (i.e., language, literacy, and control variables) and outcome variables (i.e., language tests and the social-emotional skills) of the 202 children are summarized in Table 1. In terms of home Mandarin and English language and reading environment, children varied substantially in language input, output, and material availability. On average, children received 2.39 h of Mandarin input (*SD* = 2.17) and 3.16 English input (*SD* = 2.79) at home from family members. They spoke 2.04 h of Mandarin (*SD* = 2.09) and 2.67 h of English (*SD* = 2.47) to family members. Most children started to speak Mandarin at the age of two (*SD* = 11.53 months) and started to speak English earlier, at the age of 1 year and 10 months old (*SD* = 11.73 months). Children’s mothers usually commanded the levels of Mandarin and English that allowed them to carry out a conversation. The children in the current study reported seldom visiting a library, going there once every 2 weeks on average (*SD* = 0.76). The families have on average 10–30 Mandarin books (*SD* = 1.33), 30 to 60 English books (*SD* = 1.34). They read in Mandarin two times per week (*SD* = 1.82), and read in English three times per week (*SD* = 2.01). In terms of receptive language skills, children’s Mandarin vocabulary size was 34.83 (*SD* = 9.76) and their English vocabulary size was 43.41 (*SD* = 8.05). Their Mandarin grammar score was 35.91 (*SD* = 10.96) and their English grammar score 41.31 (*SD* = 15.99).

**TABLE 1:** Descriptivein statistics and paired *t*-tests of the predictors and the outcome variables.

	***N***	**Mandarin *M (SD)***	**English *M (SD)***	***t***	***p***
**Home language exposure**					
Current input	201	2.39(2.17)	3.16 (2.79)	−2.94	0.00
Current output	199	2.04(2.09)	2.67 (2.47)	−2.53	0.01
Cumulative output	202	24.35(11.53)	22.45 (11.73)	1.84	0.07
Maternal proficiency	202	3.15(0.90)	3.00 (0.93)	2.07	0.04
**Home reading activities**					
Library visit	202	0.97(0.76)	0.97 (0.76)		
Book number	202	1.94(1.33)	2.53 (1.34)	−6.76	0.00
Reading frequency	202	1.91(1.82)	3.11 (2.01)	−7.46	0.00
**Language proficiency**					
Vocabulary	202	34.83(9.76)	43.41 (8.05)	−9.67	0.00
Grammar	202	35.91(10.96)	41.31 (15.99)	−4.99	0.00
	*N*	*M (SD)*			
**Control variables and social-emotional and behavioral skills**					
Maternal education	202	5.18 (1.29)			
Non-verbal intelligence	202	20.14 (4.65)			
Short-term memory	202	19.88 (4.37)			
Prosocial skills	202	6.89 (1.84)			
Difficulty level	198	9.69 (4.84)			

*Current input, the number of hours that family members on average speak to children in Mandarin per day; Current output, the number of hours on average that children speak to family members in Mandarin per day; Cumulative output, the onset age that children speak Mandarin; Maternal proficiency, mothers’ self-rated proficiency in Mandarin on a 1–4 point scale; Library visit, the frequency of visiting a library on a 1–6 point scale; Book number, the number of Mandarin books at home on a 1–7 point scale; Reading frequency, the frequency of reading Mandarin books at home on a 0–7 point scale; Vocabulary, receptive vocabulary size measured by BLAB; Grammar, receptive grammar measured by MGRT and TROG; Maternal education, mothers’ highest educational level; Non-verbal Intelligence, non-verbal IQ score as a measure of analytic reasoning using Raven’s; Short-term memory, short-term phonological memory score based on digit span and non-word repetition; Prosocial skills, prosocial level measured by SDQ; Difficulty level, social emotional difficulty level measured by SDQ.*

The fundamental assumption of the current study is that Singapore preschoolers’ bilingual language environment is “disharmonious,” as children’s English learning environment and outcomes are substantially better than that of English. Paired sample *t*-tests have been used to investigate the differences between the two languages, and the results confirmed the assumption that English environment was significantly better than Mandarin environment in terms language input, output, and reading activities. Children started to speak in English earlier and the difference was nearly significantly (*p* = 0.07). Regarding to children’s receptive skills, their English vocabulary and grammar were substantially better than their skills in Mandarin. Family members’ reluctance of using Mandarin might not due to their lower proficiency. For instance, mother’s Mandarin proficiency was significantly higher than that in English, suggesting that the choice of using more English was out of personal preference but not base on language competence.

In order to avoid multicollinearity, non-parametric Spearman’s correlations were used to demonstrate the relationship between the Mandarin predictors and the control variables (Table 2). Highly correlated variables (*r* = 0.6–0.9) would be averaged or excluded. Current home Mandarin input was found to be significantly and highly correlated with current home Mandarin output (*r* = 0.89). Therefore, the values of the two variables were averaged into one score (i.e., current input-output) to indicate general Mandarin usage at home every day. The other predictors are only weakly or moderately correlated, therefore, they have been included in the final analysis.

**TABLE 2:** Pearson correlations of the Mandarin predictors and the control variables.

	**1**	**2**	**3**	**4**	**5**	**6**	**7**	**8**	**9**
1. Current input	1.00								
2. Current output	0.89^∗∗^	1.00							
3. Cumulative output	–0.20^∗∗^	0.29^∗∗^	1.00						
4. Maternal proficiency	0.10	0.16^*^	–00.14	1.00					
5. Library visit	0.08	0.08	–0.08	–0.03	1.00				
6. Book number	–0.02	0.04	−0.18^*^	0.14^*^	0.14	1.00			
7. Reading frequency	0.13	0.16^*^	–0.23^∗∗^	0.18^*^	0.30^∗∗^	0.51^∗∗^	1.00		
8. Maternal education	−0.16^*^	–0.09	–0.11	0.1^∗∗^	0.11	0.23^∗∗^	0.12	1	
9. Non-verbal intelligence	–0.20^∗∗^	−0.16^*^	–0.02	0.17^*^	0.02	0.21^∗∗^	0.14	0.18^*^	1
10. Short-term memory	–0.01	0.07	−0.15^*^	0.12	0.15^*^	0.19^*^	0.13	0.20^∗∗^	0.09

^*^p < 0.05, ^∗∗^*p* < 0.01.

### Home Mandarin Environment and Receptive Mandarin Language Outcomes

Table 3 shows the results of the first research question. Children’s Mandarin receptive vocabulary and receptive grammar were predicted by home Mandarin language exposure (i.e., current Mandarin usage at home, children’s onset age of Mandarin speaking, and mother’s language proficiency), home Mandarin reading activities (i.e., number of Mandarin books at home, reading frequency, and library visits), and other individual differences (i.e., non-verbal intelligence, phonological memory, gender, and mother’s educational level). More years of using Mandarin, and higher mother’s Mandarin proficiency are positively and significantly related to both children’s Mandarin receptive vocabulary and grammar. More Mandarin input and output in the current family environment is positively and significantly associated with children’s receptive vocabulary. In terms of Mandarin reading activities, larger number of Mandarin books is significantly associated with children’s larger Mandarin receptive vocabulary, while more library visits are significantly related to children’s better Mandarin receptive grammar. Besides these home language and literacy variables, children’s individual differences in gender, and cognitive factors matter. Those children with better short-term phonological memory and non-verbal intelligence tend to have higher Mandarin receptive vocabulary and grammar proficiency. Moreover, girls have better receptive grammar than boys. In total, the model explained 40.3% of the variance in children’s Mandarin receptive vocabulary and 38% in children’s Mandarin receptive grammar, with good model-fit statistics [X^2^(22) = 34.22, *p* = 0.047, CFI = 0.97, TLI = 0.91, RMSEA = 0.05].

**TABLE 3:** The results of structural equation modeling on receptive language outcomes in Mandarin.

	**Path**	**Standardized coefficient**	**SE**	***P***
Home Mandarin language exposure	Current input-output - > Mandarin vocabulary	0.20	0.27	^∗∗∗^
	Current input-output - > Mandarin grammar	0.07	0.31	0.22
	Cumulative output - > Mandarin vocabulary	−0.32	0.05	^∗∗∗^
	Cumulative output - > Mandarin grammar	−0.30	0.06	^∗∗∗^
	Mother proficiency - > Mandarin vocabulary	0.16	0.60	^∗∗^
	Mother proficiency - > Mandarin grammar	0.17	0.68	^∗∗^
Home Mandarin reading activities	Library visits - > Mandarin vocabulary	0.08	0.71	0.18
	Library visits - > Mandarin grammar	0.12	0.81	^*^
	Book number - > Mandarin vocabulary	0.19	0.46	^∗∗^
	Book number - > Mandarin grammar	0.10	0.53	0.13
	Reading days - > Mandarin vocabulary	0.06	0.35	0.35
	Reading days - > Mandarin grammar	0.04	0.40	0.55
Control factors	Phonological memory - > Mandarin vocabulary	0.17	0.12	^∗∗^
	Phonological memory - > Mandarin grammar	0.26	0.14	^∗∗∗^
	Non-verbal intelligence - > Mandarin vocabulary	0.19	0.11	^∗∗∗^
	Non-verbal intelligence - > Mandarin grammar	0.26	0.13	^∗∗∗^
	Mother education - > Mandarin vocabulary	0.02	0.43	0.77
	Mother education - > Mandarin grammar	0.00	0.49	0.94
	Gender - > Mandarin vocabulary	0.07	1.05	0.18
	Gender - > Mandarin grammar	0.11	1.19	^*^

^∗∗∗^*p* < 0.001, ^∗∗^*p* < 0.01, ^*^p < 0.05. X^2^(22) = 34.22, *p* = 0.047, CFI = 0.97, TLI = 0.91, RMSEA = 0.05.

### Home Mandarin Environment and Children’s Social-Emotional and Behavioral Skills

Table 4 shows the results of the second research question. Children’s difficulty level and prosocial skills were predicted by home Mandarin language exposure (i.e., current Mandarin usage at home, children’s onset age of Mandarin speaking, and mother’s language proficiency), home Mandarin reading activities (i.e., number of Mandarin books at home, reading days, and library visits), and control variables (i.e., children’s gender and mother’s educational level). Children’s difficulty level was negatively and significantly related to home Mandarin literacy activities (i.e., library visits and Mandarin reading days per week at home). On the other hand, children’s prosocial skills were positively and significantly related to the number of reading days at home. Beside these factors, children’s gender was found to influence social-emotional skills. Compared with boys, girls demonstrated better prosocial skills and lower difficulty level. In total, the model explained 13% of the variance in children’s prosocial skills and 14% of the variance in children’s difficulty level. CFI, TLI, and RMSEA of the model were consistent with the cutoff model-fit criteria (i.e., CFI = 1, TLI = 1.09, and RMSEA = 0.00).

**TABLE 4:** The results of structural equation modeling on social emotional skills.

	**Path**	**Standardized coefficient**	**SE**	***P***
Home Mandarin language exposure	Current input-output - > Difficulty	0.13	0.17	0.06
	Current input-output - > Prosocial	−0.06	0.06	0.38
	Cumulative output - > Difficulty	0.04	0.03	0.60
	Cumulative output - > Prosocial	−0.01	0.01	0.84
	Mother proficiency - > Difficulty	0.02	0.37	0.81
	Mother proficiency - > Prosocial	−0.10	0.14	0.14
Home Mandarin reading activities	Library visits - > Difficulty	−0.18	0.44	^∗∗^
	Library visits - > Prosocial	0.05	0.17	0.51
	Book number - > Difficulty	−0.11	0.11	0.15
	Book number - > Prosocial	0.09	0.29	0.27
	Reading days - > Difficulty	−0.20	0.22	^*^
	Reading days - > Prosocial	0.32	0.08	^∗∗∗^
Control factors	Mother education - > Difficulty	−0.10	0.26	0.14
	Mother education - > Prosocial	0.05	0.10	0.45
	Gender - > Difficulty	−0.18	0.65	^∗∗^
	Gender - > Prosocial	0.18	0.24	^∗∗^

^∗∗∗^*p* < 0.001, ^∗∗^*p* < 0.01, ^*^p < 0.05. X^2^(7) = 5.182, *p* = 0.64, CFI = 1, TLI = 1.09, RMSEA = 0.00.

## Discussion

The current study examined how home language exposure and reading activities affect English-Mandarin bilingual children’s Mandarin language competence and social-emotional and behavioral skills. The study intends to concretize the recent proposed conceptual framework “harmonious bilingual development” (De Houwer, 2015) by decomposing home language and literacy environment and closely examining its impact on child bilinguals’ MTL competence and social-emotional and behavioral skills. The results demonstrated that home MTL environment plays a crucial role in children’s language proficiency and social competence, and pinpointed in which specific aspects parents could make efforts in order to change the environment. For children’s Mandarin receptive language competence, both home language environment (i.e., current amount of language input and output, cumulative language output, mother’s language proficiency), and home reading activities (i.e., number of Mandarin books and school library) play a role. In contrast, for children’s social-emotional and behavioral skills, home reading activities were found to be more important (i.e., reading frequency and library visits) than language exposure.

### The Potential Impact of Home Language Exposure on MTL Learning

Our results confirmed the previous findings that MTL exposure is crucial in children’s MTL maintenance (e.g., Dixon et al., 2012, in the context of Singapore; Hammer et al., 2009; Hoff et al., 2012, in the context of the United States), and that it may influence multiple language domains, such as vocabulary, and grammar. Children are predicted to have higher MTL receptive skills if they have a higher MTL input amount, higher input quality, and sufficient length of time to practice the language. The translation of the finding is that parents with good MTL proficiency should be encouraged to use the language with their children as much as they can, and they should also consider creating more opportunities for their children to use the language. Otherwise, a limited amount of exposure may hinder their child’s MTL learning. To take Singapore as an example, the local parents prefer to speak English to their children at home out of utility concerns and rely on MTL instruction at school to develop their child’s MTL proficiency. Nevertheless, MTL is a subject being taught at the preschool level with the class only lasting 40 min to 1 h per day on average, suggesting that fully relying on the school exposure might not be sufficient. Our results, together with the findings of Dixon et al. (2012) and Sun et al. (forthcoming) provide consistent evidence that parents should be more aware of their significant role in their children’s MTL learning. A slight modification of their home language policy may have a substantial impact on their child’s MTL outlook. Bornstein et al. (1998) argued that children’s language development requires bottom-up resources to nourish its growth. Frequent interaction with other interlocutors at home would greatly contribute to the flourishing of the target language system.

### The Potential Impact of Home Reading Activities on Receptive MTL Outcomes and Social-Emotional and Behavioral Skills

Compared to home language exposure, there are much fewer studies on the influence of home MTL literacy experience on child bilinguals’ receptive vocabulary and grammar skills in MTL. The current finding strengthens previous studies that demonstrate a connection between the extent of home reading activities (without distinguishing the language used in the reading) and the level of children’s receptive MTL skills (Bohman et al., 2010; Hammer et al., 2012; Farver et al., 2013; Lewis et al., 2016). By targeting MTL reading activities *per se*, the current study managed to establish a direct link between the literacy experience, and the language outcome in the same language of the child bilinguals. The association between reading activities (i.e., library visits and the number of Mandarin language books at home) and children’s receptive Mandarin language proficiency might be explained by the books’ complicated and diverse lexical and syntactic structures (Montag et al., 2015). To take vocabulary as an example, preschoolers’ books contain 16.3 complex words per 1000 words, which is five times the amount of sophisticated words as are used in common conversation (Hayes and Ahrens, 1988). Weizman and Snow (2001) found that that the use of these rare words matters and the frequency of a mother’s use of such sophisticated words with her 5 year old child could predict the child’s vocabulary performance in both kindergarten and second grade at primary school. During book reading, children may need to answer parents’ questions and would be asked to retell the story. Such interaction would elicit more output from children in the target language and facilitate their language production. Meta-analyses have revealed that early book reading experiences have a lasting effect on child language development, being able to explain 8% of monolingual children’s language and reading performance (Bus et al., 1995), and 12% of children’s differences in oral language skills (Mol and Bus, 2011).

Regarding the relations between home environment and children’s social-emotional skills, we have found reading frequency and library visits are negatively and significantly associated with children’s difficulty level, and Mandarin language reading frequency at home is positively related to children’s prosocial skills. Reading activities seem to function as a protection scheme for children’s early social-emotional wellbeing, in line with previous findings (Farver et al., 2006, 2013; Aram and Shapira, 2012; Aram et al., 2017). The benefits might be attributed to both the content of the books and the nature of the communication between child and adults during reading sessions (Aram and Shapira, 2012; Aram et al., 2017). Children’s books often contain social and emotional experiences of the protagonist(s), thus providing children rich information to comprehend social, and emotional concepts (Zeece, 2004). For instance, fairytales and folktales are usually abundant with social-emotional themes, and it is suggested that parents take advantage of these books to help children work out complex relations between mental representation and reality (Adrian et al., 2005). Besides content, the conversation between adult and child during shared book reading is also believed to promote children’s social-emotional and behavioral skills. Such conversation usually invites children to discuss the emotions, attitudes, and behaviors of the protagonists. Facilitated by the adult’s contextualized (e.g., labeling the emotion) and decontextualized (e.g., inferences) questions and comments, the dialogue would promote children to better understand the concepts and reflect on the motivations of the characters’ actions. In other words, story comprehension needs children to connect what happens in the story (i.e., the action landscape) with an analysis of the protagonists’ intentions and motivations that lie beneath their actions (“the consciousness landscape”) (Bruner, 1990). This learning process would help children to comprehend, express, and regulate their own behaviors and emotions. Besides learning from the storybook, shared book reading itself provides a good platform for parents and children to establish positive interactions, which would foster children’s emotional understanding. A delightful shared book reading routine would generate positive feelings from both the parent and the child (Bus and van Ijzendoorn, 1997), encouraging children to explore their surroundings and to recognize, and regulate emotions (Denham et al., 2003; Aram and Shapira, 2012).

Our findings suggest that parents should be informed that their home literacy behaviors may not only affect children’s receptive language skills in MTL, but also children’s social-emotional wellbeing. Such reading activities are usually within the parent’s capacity, therefore, parents should be encouraged to conduct these activities in the home setting. Our data revealed that on average family members only read two times per week to children (*M* = 1.91, *SD* = 1.82), and each household has approximately 10–30 Mandarin children’s books (*M* = 1.94, *SD* = 1.33). Visits to the public library to read Mandarin children’s books did not occur often, happening once every 2 weeks on average (*M* = 0.97, *SD* = 0.76). The results suggest that regular but not necessarily frequent reading practice would impact children’s MTL and wellbeing profiles. Moreover, a family does not need to have many children’s MTL books to benefit from these readings. These findings indicate that a decent MTL reading environment is affordable for parents with busy schedules and families with low household income.

## Conclusion and Implications

There are three major limitations of the study. First, this is a cross-sectional study, therefore, only the relations between home environment and children’s language and social competence could be inferred. Future studies could follow the participants longitudinally and examine possible variations in the trajectory of the relations. An experiment (e.g., balanced bilingual vs. one language dominant bilingual) could be carried out to explore the causality in the association. Second, the current study only considers parents’ evaluation of children’s social-emotional and behavioral skills with one questionnaire (i.e., SDQ). Due to the multifaceted nature of children’s social-emotional wellbeing, it would be better to engage multiple stakeholders (e.g., preschool teachers) with various measures (e.g., NEPSY-II) to triangulate the observation. Third, the current study focuses on children’s receptive Mandarin language skills and the future studies might consider include productive language measures as well, to holistically examine the impact of home language, and literacy environment on children’s MTL learning. The author did examine children’s Mandarin semantic fluency skills in the current study, however, considering the substantial association of the task with children’s executive functions, children’s semantic fluency has not been taken into the final model for analysis. If taken into the model, the result demonstrated that children’s semantic fluency in Mandarin has been affected in the same way as the receptive knowledge was, implying that home language and literacy environment might exert the similar influence on children’s productive language skills. Future studies are suggested to test child bilingual’s productive language skills with standardized tests (e.g., expressive one word vocabulary test) to verify this hypothesis.

Despite these limitations, the study demonstrates the positive role that parents could play in children’s MTL and social-emotional wellbeing. Parental literacy practice at home may not only affect children’s Mandarin proficiency, but also benefit children’s social-emotional and behavioral skills. As Aram et al. (2017) advocated, policy makers and educational professionals should consider parental involvement in children’s general wellbeing as a crucial goal of early childhood education. Tutorials should be provided to the public, to instruct them on how to use home reading activities to engage children in a discussion and to explore social issues in related decontextualized talk. Preschool educators should receive guidance on how to encourage parents to conduct readings at home. Such implementation of the research findings would allow researchers, parents, educators, and policy makers to work together to advance children’s early MTL learning and social-emotional wellbeing.

## Data Availability

The datasets for this study will not be made publicly available because of the privacy contract with the participants.

## Ethics Statement

This study was carried out in accordance with the recommendations of the IRB-2017-04-019, Nanyang Technological University Institutional Review Board. The protocol was approved by the Nanyang Technological University Institutional Review Board. All subjects gave written informed consent in accordance with the Declaration of Helsinki.

## Author Contributions

HS carried out the work, analyzed the data, interpreted the results, and wrote the manuscript.

## Conflict of Interest Statement

The author declares that the research was conducted in the absence of any commercial or financial relationships that could be construed as a potential conflict of interest.

## References

[B1] Aarts R. Ronde S. (2006). Tweetalig onderwijs met vervroegd Engels in het basisonderwijs. *Levende Talen Tijdschrift* 7 3–14.

[B2] Adrian J. E. Clemente R. A. Villanueva L. Rieffe C. (2005). Parent–child picture-book reading, mothers’ mental state language and children’s theory of mind. *J. Child Lang.* 32 673–686. 10.1017/S0305000905006963 16220639

[B3] Aram D. Aviram S. (2009). Mothers’ storybook reading and kindergartners’ socioemotional and literacy development. *Read. Psychol.* 30 175–194. 10.1080/02702710802275348

[B4] Aram D. Deitcher D. B. Shoshan T. S. Ziv M. (2017). Shared book reading interactions within families from low socioeconomic backgrounds and children’s social understanding and prosocial behavior. *J. Cogn. Educ. Psychol.* 16 157–177. 10.1891/1945-8959.16.2.157

[B5] Aram D. Shapira R. (2012). Parent-child shared book reading and children’s language, literacy, and empathy development. *Riv. Ital. Educ. Fam.* 2 55–65.

[B6] Aryadoust V. Liu S. (2015). Predicting EFL writing ability from levels of mental representation measured by Coh-Metrix: a structural equation modeling study. *Assess. Writ.* 24 35–58. 10.1016/j.asw.2015.03.001

[B7] Australian Institute of Health and Welfare (2012). *Social and Emotional Wellbeing: Development of a Children’s Headline Indicator. Cat. No. PHE 158.* Canberra: AIHW.

[B8] Baker C. Wright W. E. (2017). *Foundations of bilingual education and bilingualism, Bilingual education & bilingualism: 106.* Bristol, U.K: Multilingual Matters.

[B9] Bishop D. (2003). *Test for Reception of Grammar*, 2nd Edn London: Pearson Assessment.

[B10] Bohman T. M. Bedore L. M. Peña E. D. Mendez-Perez A. Gillam R. B. (2010). What you hear and what you say: language performance in Spanish English Bilinguals. *Int. J. Biling. Educ. Bilingual.* 13 325–344. 10.1080/13670050903342019 21731899 PMC3128885

[B11] Bornstein M. H. Haynes M. O. Painter K. M. (1998). Sources of child vocabulary competence: a multivariate model. *J. Child Lang.* 25 367–393. 10.1017/s0305000998003456 9770912

[B12] Bruner J. S. (1990). *Act of Meaning.* Cambridge. MA: Harvard University Press.

[B13] Bull R. Lee K. Koh I. H. C. Poon K. K. L. (2016). Confirmatory factor analysis of the strengths and difficulties questionnaire in Singaporean kindergartners. *Child Care Health Dev.* 42 109–116. 10.1111/cch.12288 26470606

[B14] Burgess S. (2005). The preschool home literacy environment provided by teenage mothers. *Early Child Dev. Care* 175 249–258. 10.1080/0300443042000266303

[B15] Burgess S. R. Hecht S. A. Lonigan C. J. (2002). Relations of the home literacy environment (HLE) to the development of reading-related abilities: a one-year longitudinal study. *Read. Res. Q.* 37 408–426. 10.1598/rrq.37.4.4

[B16] Bus A. G. van Ijzendoorn M. H. (1997). Affective dimension of mother-infant picturebook reading. *J. School Psychol.* 35 47–60. 10.1016/S0022-4405(96)00030-1

[B17] Bus A. G. Van IJzendoorn M. H. Pellegrini A. D. (1995). Joint book reading makes for success in learning to read: a meta-analysis on intergenerational transmission of literacy. *Rev. Educ. Res.* 65 1–21. 10.3102/00346543065001001

[B18] Cavallaro F. Ng B. C. (2014). “Language in Singapore: From Multilingualism to English Plus,” in *Challenging the Monolingual Mindset*, eds Hajek J. Slaughter Y. (Bristol: Multilingual Matters), 33–48. 10.21832/9781783092529-005

[B19] Chondrogianni V. Marinis T. (2011). Differential effects of internal and external factors on the development of vocabulary, tense morphology and morpho-syntax in successive Bilingual children. *Linguist. Appr. Bilingual.* 1 318–345. 10.1075/lab.1.3.05cho

[B20] Collins B. A. Toppelberg C. O. Suárez-Orozco C. O’Connor E. Nieto-Castañon A. (2011). Cross-sectional associations of Spanish and English competence and well-being in Latino children of immigrants in kindergarten. *Int. J. Sociol. Lang.* 2011 5–23.

[B21] Croft S. Stride C. Maughan B. Row R. (2015). Validity of the strengths and difficulties questionnaire in preschool-aged children. *Pediatrics* 5:1210. 10.1542/peds.2014-2920 25847804

[B22] Cummins J. (2001). Bilingual children’s mother tongue: why is it important for education? *Sprogforum* 19 15–20.

[B23] Cummins J. (2005). A proposal for action: strategies for recognizing heritage language competence as a learning resource within the mainstream classroom. *Mod. Lang. J.* 89 585–592.

[B24] Daller M. Ongun Z. (2017). The Threshold Hypothesis revisited: bilingual lexical knowledge and non-verbal IQ development. *Int. J. Bilingual.* 22 675–694. 10.1177/1367006917690835

[B25] De Houwer A. (2007). Parental language input patterns and children’s bilingual use. *Appl. Psycholinguist.* 283 411–424. 10.1017/s0142716407070221 12846302

[B26] De Houwer A. (2009). *Bilingual First Language Acquisition, MM Textbooks: 2.* Bristol: Multilingual Matters.

[B27] De Houwer A. (2015). Harmonious bilingual development: Young families’ well-being in language contact situations. *Int. J. Bilingual.* 19 169–184. 10.1177/1367006913489202

[B28] Denham S. A. Blair K. A. DeMulder E. Levitas J. Sawyer K. Auerbach-Major S. (2003). Preschool emotional competence: pathway to social competence? *Child Dev.* 74 238–256. 10.1111/1467-8624.00533 12625448

[B29] Dickinson D. K. Tabors P. O. (2001). *Beginning Literacy With Language: Young Children Learning at Home and School.* Baltimore, MD: Brookes Publishing Company.

[B30] Dixon L. Q. Zhao J. Quiroz B. G. Shin J. Y. (2012). Home and community factors influencing bilingual children’s ethnic language vocabulary development. *Int. J. Bilingual.* 16 541–565. 10.1177/1367006911429527

[B31] Donaldson J. A. Loh J. Mudaliar S. Kadir M. M. Wu B. Q. Yeoh Y. L. (2013). Measuring poverty in Singapore: frameworks for consideration. *Soc. Space* 6 58–66.

[B32] Downs A. Strand P. S. Heinrichs N. Cerna S. (2012). Use of the teacher version of the strengths and difficulties questionnaire with German and American preschoolers. *Early Educ. Dev.* 23 493–516. 10.1080/10409289.2010.532082

[B33] Dunn L. M. Dunn D. M. (2007). *Peabody Picture Vocabulary Test*, 4th Edn Minneapolis: Pearson.

[B34] Ebert S. Lockl K. Weinert S. Anders Y. Kluczniok K. Rossbach H. (2013). Internal and external influences on vocabulary development in preschool children. *School Effective. School Improve.* 24 138–154. 10.1111/j.1467-8624.2010.01539.x 21291427 PMC3058312

[B35] Eisenberg N. (2006). “Prosocial Behavior,” in *Children’s Needs III: Development, Prevention, and Intervention*, eds Bear G. G. Minke K. M. (Washington, DC: National Association of School Psychologists), 313–324.

[B36] Eriksson M. Marschik P. B. Tulviste T. Almgren M. Pereira M. P. Wehberg S. (2012). Differences between girls and boys in emerging language skills: evidence from 10 language communities. *Br. J. Dev. Psychol.* 30 326–343. 10.1111/j.2044-835X.2011.02042.x 22550951

[B37] Farver J. A. M. Xu Y. Y. Eppe S. Lonigan C. J. (2006). Home environments and young Latino children’s school readiness. *Early Child. Res. Q.* 21 196–212. 10.1016/j.ecresq.2006.04.008

[B38] Farver J. A. M. Xu Y. Y. Lonigan C. J. Eppe S. (2013). The home literacy environment and latino head start children’s emergent literacy skills. *Dev. Psychol.* 4:775. 10.1037/a0028766 22662767

[B39] Goh Y. S. Ng C. W. (2015). Hard truths about Chinese language policy and planning in Singapore. *Glob. Chin.* 1 267–279.

[B40] Goodman R. (1997). The strengths and difficulties questionnaire: a research note. *J. Child Psychol. Psychiatry* 38 581–586. 10.1111/j.1469-610.1997.tb01545.x 9255702

[B41] Gopinathan S. (2004). Language planning in education in Singapore: history, transitions, futures. *Review* 12 14–23.

[B42] Gudykunst W. B. Ting-Toomey S. (1990). “Ethnic identity, language and communication breakdowns,” in *Handbook of Language and Social Psychology*, eds Giles H. Robinson W. P. (Oxford: John Wiley & Sons), 309–327.

[B43] Hammer C. S. Davison M. D. Lawrence F. R. Miccio A. W. (2009). The effect of maternal language on bilingual children’s vocabulary and emergent literacy development during head start and kindergarten. *Sci. Stud. Read.* 13 99–121. 10.1080/10888430902769541 23606802 PMC3627728

[B44] Hammer C. S. Hoff E. Uchikoshi Y. Gillanders C. Castro D. C. Sandilos L. E. (2014). Review: the language and literacy development of young dual language learners: a critical review. *Early Child. Res. Q.* 29 715–733. 10.1016/j.ecresq.2014.05.008 25878395 PMC4394382

[B45] Hammer C. S. Komaroff E. Rodriguez B. L. Lopez L. M. Scarpino S. E. Goldstein B. (2012). Predicting Spanish–english Bilingual children’s language abilities. *J. Speech Lang. Hear Res.* 55 1251–1264. 10.1044/1092-4388(2012/11-0016) 22337497 PMC3524528

[B46] Han W. J. (2010). Bilingualism and socioemotional well-being. *Child. You. Serv. Rev.* 32 720–731. 10.1016/j.childyouth.2010.01.009 26704302

[B47] Hartas D. (2011). Families’ social backgrounds matter: socio−economic factors, home learning and young children’s language, literacy and social outcomes. *Br. Educ. Res. J.* 37 893–914. 10.1080/01411926.2010.506945

[B48] Hayes D. P. Ahrens M. G. (1988). Vocabulary simplification for children: a special case of motherese. *Child Lang.* 15 135–169. 10.1017/s0305000900012411 3209647

[B49] Hess R. D. Holloway S. D. (1984). “Family and school as educational institutions,” in *Review of Child Development Research*, ed. Parke R. D. (Chicago, IL: University of Chicago Press), 179–222.

[B50] Hoff E. (2006). How social contexts support and shape language development. *Dev. Rev.* 26 55–88. 10.1016/j.dr.2005.11.002

[B51] Hoff E. Core C. Place S. Rumiche R. SeÑOr M. Parra M. (2012). Dual language exposure and early bilingual development. *J. Child Lang.* 39 1–27. 10.1017/S0305000910000759 21418730 PMC4323282

[B52] Jia G. Aaronson D. (2003). A longitudinal study of chinese children and adolescents learning english in the United States. *Appl. Psycholinguist.* 24 131–161. 10.1017/s0142716403000079

[B53] Johnson A. D. Martin A. Brooks-Gunn J. Petrill S. A. (2008). Order in the house!: associations among household chaos, the home literacy environment, maternal reading ability, and children’s early reading. *Merrill-Palmer Q.* 54 445–472. 10.1353/mpq.0.0009 19526070 PMC2695402

[B54] Kahn R. Kellner D. (2005). Reconstructing technoliteracy: a multiple literacies approach. *E-Learn. Digit. Media* 2 238–251. 10.2304/elea.2005.2.3.4

[B55] Kenny D. A. McCoach D. B. (2003). Effect of the number of variables on measures of fit in structural equation modeling. *Struct. Equa. Model. Multidis. J.* 10 333–351. 10.1207/S15328007SEM1003_1

[B56] Klem L. (2000). “Structural equation modeling,” in *Reading and Understanding MORE Multivariate Statistics*, ed. Yarnold P. R. (Washington, DC: American Psychological Association).

[B57] Kline R. B. (2011). *Principles and Practice of Structural Equation Modeling*, 3rd Edn New York, NY: The Guilford Press.

[B58] Lantolf J. P. Thorne S. L. (2007). “Sociocultural Theory and Second Language Learning,” in *Theories in Second Language Acquisition*, eds van Patten B. Williams J. (Mahwah, NJ: Lawrence Erlbaum).

[B59] Lewis K. Sandilos L. E. Hammer C. S. Sawyer B. E. Mendez L. I. (2016). Relations among the home language and literacy environment and children’s language abilities: a study of head start dual language learners and their mothers. *Early Educ. Dev.* 27 478–494. 10.1080/10409289.2016.1082820 27429533 PMC4941823

[B60] Li L. Tan C. L. (2016). Home literacy environment and its influence on Singaporean children’s Chinese oral and written language abilities. *Early Childhood Educ. J.* 44 381–387. 10.1007/s10643-015-0723-4

[B61] McLoyd V. C. (1998). Socioeconomic disadvantage and child development. *Am. Psycholog.* 53 185–204. 10.1037/0003-066X.53.2.185 9491747

[B62] Mol S. E. Bus A. G. (2011). To read or not to read: a meta-analysis of print exposure from infancy to early adulthood. *Psychol. Bull.* 137 267–296. 10.1037/a0021890 21219054

[B63] Montag J. L. Jones M. N. Smith L. B. (2015). The words children hear: picture books and the statistics for language learning. *Psychol. Sci.* 26 1489–1496. 10.1177/0956797615594361 26243292 PMC4567506

[B64] Montrul S. (2010). Current issues in heritage language acquisition. *Ann. Rev. Appl. Linguist.* 30 3–23. 10.1017/s0267190510000103

[B65] Niclasen J. Teasdale T. W. Andersen A. N. Skovgaard A. M. Elberling H. Obel C. (2012). Psychometric properties of the Danish strength and difficulties questionnaire: the SDQ assessed for more than 70,000 raters in four different cohorts. *PLoS One* 7:e032025. 10.1371/journal.pone.0032025 22384129 PMC3288071

[B66] Oller D. K. Jarmulowicz L. (2007). “Language and literacy in bilingual children in the early school years,” in *Blackwell Handbook of Language Development*, eds Hoff E. Shatz M. (Malden, MA: Blackwell Publishing), 368–386. 10.1002/9780470757833.ch18

[B67] Oon C. Kor K. K. (2009). *Was Chinese Wrongly Taught for Thirty Years? The Straits Times.* Available at: https://www.asiaone.com/News/Education/Story/A1Story20091128-182805.html (accessed July 4, 2019).

[B68] Paradis J. (2011). Individual differences in child English second language acquisition: comparing child-internal and child-external factors. *Linguist. Approach. Bilingual.* 1 213–237. 10.1075/lab.1.3.01par

[B69] Paradis J. Nicoladis E. Crago M. Genesee F. (2011). Bilingual children’s acquisition of the past tense: a usage-based approach. *J. Child Lang.* 38 554–578. 10.1017/S0305000910000218 20738891

[B70] Pearson B. Z. (2007). Social factors in childhood bilingualism in the United States. *Appl. Psycholinguist.* 28 399–410. 10.1017/s014271640707021x

[B71] Portes A. Rumbaut R. (2001). *Legacies: The Story of Immigrant Second Generation.* Berkeley, CA: University of California Press.

[B72] Raven J. Rust J. (2004). *Coloured Progressive Matrices and Crichton Vocabulary Scale.* London: Pearson.

[B73] Raver C. C. Knitzer J. (2002). *Ready to Enter: What Research Tells Policymakers About Strategies to Promote Social and Emotional School Readiness Among Three-and Four-Year-Olds. Policy Paper 3.* Columbia: Columbia University, National Center for Children in Poverty.

[B74] Ren Y. G. Wyver S. Rattanasone N. X. Demuth K. (2016). Social competence and language skills in mandarin–english bilingual preschoolers: the moderation effect of emotion regulation. *Early Educ. Dev.* 27 303–317. 10.1080/10409289.2015.1066639

[B75] Scheele A. F. Leseman P. P. Mayo A. Y. (2010). The home language environment of monolingual and bilingual children and their language proficiency. *Appl. Psycholinguist.* 31 117–140. 10.1371/journal.pone.0209981 30653525 PMC6336237

[B76] Singapore Department of Statistics (2017). *Population Trends 2017.* Available at: https://www.singstat.gov.sg/-/media/files/publications/population/population2017.pdf (accessed August 14, 2018).

[B77] Stone L. L. Otten R. Engels R. C. Vermulst A. A. Janssens J. M. (2010). Psychometric properties of the parent and teacher versions of the strengths and difficulties questionnaire for 4- to 12-year-olds: a review. *Clin. Child Fam. Psychol. Rev.* 13 254–274. 10.1007/s10567-010-0071-2 20589428 PMC2919684

[B78] Sun H. Steinkrauss R. Tendeiro J. de Bot K. (2016). Individual differences in very young children’s English acquisition in China: internal and external factors. *Bilingual. Lang. Cogn.* 19 550–566. 10.1017/S1366728915000243

[B79] Sun H. Steinkrauss R. Wieling M. de Bot K. (2018a). Individual differences in very young Chinese children’s English vocabulary breadth and semantic depth: internal and external factors. *Int. J. Biling. Educ. Bilingual.* 21 405–425. 10.1080/13670050.2016.1178706

[B80] Sun H. Yin B. Amsah F. O’Brien B. A. (2018b). Differential effects of internal and external factors in early bilingual vocabulary learning: the case of Singapore. *Appl. Psycholinguist.* 39 383–411. 10.1017/S014271641700039X

[B81] Sun H. Yussof N. T. B. Habib Mohamed M. B. B. Rahim A. B. Bull B. Cheung M. W. L. (2018c). Bilingual language experience and children’s social-emotional and behavioral skills: a cross-sectional study of Singapore preschoolers. *Int. J. Bilingual Educ. Bilingual.* 1–16. 10.1080/13670050.2018.1461802

[B82] Sun H. Ng S. C. O’Brien B. A. Fritzsche T. (forthcoming). *Child, family, and preschool. (factors)of heritage vocabulary development among bilingual children with diverse linguistic and ethnical backgrounds.*

[B83] Swain M. (1993). The output hypothesis: just speaking and writing aren’t enough. *Can. Mod. Lang. Rev.* 50 158–164. 10.3138/cmlr.50.1.158

[B84] Unsworth S. Persson L. Prins T. de Bot K. (2015). An investigation of factors affecting early foreign language learning in the Netherlands. *Appl. Linguist.* 35 527–548.

[B85] Vahedi S. Farrokhi F. Farajian F. (2012). Social Competence and Behavior problems in preschool children. *Iran. J. Psychiatry* 7 126–134. 23139694 PMC3488868

[B86] Wagner R. Torgesen J. K. Rashotte C. (1999). *Comprehensive Test of Phonological Processing.* Austin, TX: Pro-Ed.

[B87] Weizman Z. O. Snow C. E. (2001). Lexical input as related to children’s vocabulary acquisition: effects of sophisticated exposure and support for meaning. *Dev. Psychol.* 37 265–279. 10.1037/0012-1649.37.2.265 11269394

[B88] Winer A. C. Thompson R. (2013). *How Poverty and Depression Impact a Child’s Social and Emotional Competence (Policy Brief 1-10).* Davis: University of California.

[B89] Wolf E. J. Harrington K. M. Clark S. L. Miller M. W. (2013). Sample size requirements for structural equation models an evaluation of power, bias, and solution propriety. *Educ. Psychol. Measure.* 73 913–934. 10.1177/0013164413495237 25705052 PMC4334479

[B90] Yu B. (2013). Issues in bilingualism and heritage language maintenance: perspectives of minority-language mothers of children with autism spectrum disorders. *Am. J. Speech Lang. Pathol.* 22 10–24. 10.1044/1058-0360(2012/10-0078) 23071196

[B91] Zeece P. D. (2004). Promoting empathy and developing caring readers. *Early Childhood Educ. J.* 31 193–199. 10.1023/b:ecej.0000012314.00539.12

[B92] Ziv M. Smadja M. Aram D. (2013). Mothers’ mental-state discourse with preschoolers during storybook reading and wordless storybook telling. *Early Childhood Res. Q.* 28 177–186. 10.1016/j.ecresq.2012.05.005

